# Genome-Wide Association Study for Adult-Plant Resistance to Stripe Rust in Chinese Wheat Landraces (*Triticum aestivum* L.) From the Yellow and Huai River Valleys

**DOI:** 10.3389/fpls.2019.00596

**Published:** 2019-05-16

**Authors:** Li Long, Fangjie Yao, Can Yu, Xueling Ye, Yukun Cheng, Yuqi Wang, Yu Wu, Jing Li, Jirui Wang, Qiantao Jiang, Wei Li, Jian Ma, YaXi Liu, Mei Deng, Yuming Wei, Youliang Zheng, Guoyue Chen

**Affiliations:** ^1^Triticeae Research Institute, Sichuan Agricultural University, Chengdu, China; ^2^State Key Laboratory of Crop Genetics of Disease Resistance and Disease Control, Sichuan Agricultural University, Chengdu, China; ^3^College of Agronomy, Sichuan Agricultural University, Chengdu, China

**Keywords:** strip rust, adult-plant resistance, Chinese wheat landraces, genome-wide association study, Diversity Arrays Technology, simple sequence repeat

## Abstract

Stripe rust (also known as yellow rust), caused by the pathogen *Puccinia striiformis* f. sp. *tritici* (*Pst*), is a common and serious fungal disease of wheat (*Triticum aestivum* L.) worldwide. To identify effective stripe rust resistance loci, a genome-wide association study was performed using 152 wheat landraces from the Yellow and Huai River Valleys in China based on Diversity Arrays Technology and simple sequence repeat markers. Phenotypic evaluation of the degree of resistance to stripe rust at the adult-plant stage under field conditions was carried out in five environments. In total, 19 accessions displayed stable, high degrees of resistance to stripe rust development when exposed to mixed races of *Pst* at the adult-plant stage in multi-environment field assessments. A marker–trait association analysis indicated that 51 loci were significantly associated with adult-plant resistance to stripe rust. These loci included 40 quantitative trait loci (QTL) regions for adult-plant resistance. Twenty identified resistance QTL were linked closely to previously reported yellow rust resistance genes or QTL regions, which were distributed across chromosomes 1B, 1D, 2A, 2B, 3A, 3B, 4A, 4B, 5B, 6B, 7A, 7B, and 7D. Six multi-trait QTL were detected on chromosomes 1B, 1D, 2B, 3A, 3B, and 7D. Twenty QTL were mapped to chromosomes 1D, 2A, 2D, 4B, 5B, 6A, 6B, 6D, 7A, 7B, and 7D, distant from previously identified yellow rust resistance genes. Consequently, these QTL are potentially novel loci for stripe rust resistance. Among the 20 potentially novel QTL, five (*QDS.sicau-2A*, *QIT.sicau-4B*, *QDS.sicau-4B.2*, *QDS.sicau-6A.3*, and *QYr.sicau-7D*) were associated with field responses at the adult-plant stage in at least two environments, and may have large effects on stripe rust resistance. The novel effective QTL for adult-plant resistance to stripe rust will improve understanding of the genetic mechanisms that control the spread of stripe rust, and will aid in the molecular marker-assisted selection-based breeding of wheat for stripe rust resistance.

## Introduction

Wheat (*Triticum aestivum* L.) is an important food crop worldwide (Juliana et al., 2017) that is persistently threatened by attack from diverse rapidly evolving pathogens (Riaz et al., 2018). Among these biotic stresses, stripe rust caused by the pathogen *Puccinia striiformis* f. sp. *tritici* (*Pst*) is a major global threat to wheat production (Wellings, 2011; Kumar et al., 2016), especially in China (Wan et al., 2007; McIntosh et al., 2018). The five-leading wheat-producing provinces in China are Henan, Hebei, Shandong, Jiangsu, and Anhui, which contribute more than 60% of the national production (Wan et al., 2007). The first four provinces mentioned include the Yellow and Huai River Valleys, which are the main wheat-growing regions and have a unique stripe rust epidemic system (Chen and Kang, 2017). Analysis of the genetic diversity of wheat landraces from the Yellow and Huai River Valleys in China will provide information important for breeding of disease resistance in wheat.

Since the widespread stripe rust epidemic of the 1950s, extensive research has been conducted into the epidemiology and management of this disease. To date, 80 yellow rust resistance (*Yr*) genes have been permanently named in wheat, including the recently mapped *Yr79* (Feng et al., 2018) and *Yr80* (Nsabiyera et al., 2018), and 67 stripe rust resistance genes have been temporarily designated, including all-stage resistance (also termed seedling resistance) and adult-plant resistance (APR) (Wang and Chen, 2017). Although these *Yr* genes have been identified in diverse wheat accessions, the race specificity of seedling resistance genes limits their efficacy against pathotypes (Kankwatsa et al., 2017). In contrast, APR is generally considered to be durable, but APR genes represent a minority of known resistance genes (Kankwatsa et al., 2017; Yuan et al., 2018). Therefore, enhancing the resistance of adult plants to cope with evolving races of *Pst* is the preferred strategy for resistance breeding. Although traditional breeding has substantially improved wheat cultivars, the practices are time-consuming and of low efficiency (Liu W.Z. et al., 2017). However, breeding for resistance is the most cost-effective and eco-sustainable approach to prevent disease-related yield losses (Kumar et al., 2016; Juliana et al., 2018; Singh et al., 2018). The development of molecular markers linked to *Yr* genes or quantitative trait loci (QTL) can facilitate marker-assisted selection and improve the efficiency of breeding disease-resistant wheat (Miedaner and Korzun, 2012; Ayana et al., 2018).

Genome-wide association study (GWAS) shows potential advantages over traditional QTL mapping and linkage analysis, such as enhanced resolution and broader allele coverage, as well as being less time-consuming and much more cost effective (Olukolu et al., 2016). A GWAS is a powerful approach that can capture trait loci and utilize linkage disequilibrium (LD) to examine marker–trait associations (MTAs) and identify novel genes associated with complex quantitative phenotypic variation (Yang et al., 2015; Liu W.Z. et al., 2017). This technique has been successfully applied to elucidate the genetic architecture of disease resistance in a variety of plant species, such as *Arabidopsis* (Rajarammohan et al., 2018), rice (Korinsak et al., 2018), maize (Rashid et al., 2018), grain sorghum (Adeyanju et al., 2015), and soybean (Passianotto et al., 2017). In wheat, GWAS has been used to study complex agronomic traits (Liu W.Z. et al., 2017; Sun et al., 2017), leaf rust (Gao et al., 2016), and stem rust (Kankwatsa et al., 2017; Edae et al., 2018). In addition, GWAS has enabled verification of stripe rust resistance and identification of the underlying resistance genes in wheat (Juliana et al., 2018).

In this research, we used a population of 152 landraces of wheat grown in the Yellow and Huai River Valleys to address the following three objectives: (a) to evaluate the adult-plant responses to stripe rust infection in multiple environments under field conditions, (b) to assess the genetic diversity of the selected wheat landraces based on Diversity Arrays Technology (DArT) and simple sequence repeat (SSR) markers, and (c) to identify genomic regions associated with stripe rust resistance in these wheat landraces using a mixed linear model approach and to discover potential novel genes and/or QTL for stripe rust resistance.

## Materials and Methods

### Plant Materials

In total, 152 wheat landraces from the Yellow and Huai River Valleys of China were used in this study. The panel of accessions originated from five Chinese provinces, namely Shandong (52), Henan (45), Hebei (26), Shaanxi (15), and Jiangsu (14). The seeds used in this study were sourced from the Chinese Academy of Agricultural Sciences (germplasm numbers are preceded by the abbreviation ZM). Details on the landraces are provided in Supplementary Table S1.

### Genotypic Analysis

Genomic DNA was extracted from a single plant for each of the accessions using the cetyl trimethyl ammonium bromide method (Stewart and Via, 1993). Samples of genomic DNA from each accession were subjected to selective genotyping using the DArT-seq^1^ platform. All accessions were also genotyped using 135 SSR markers with 865 polymorphic allele variations, which were detected based on the published sequences of Röder et al. (1998), Pestsova et al. (2000), Sourdille et al. (2001), Somers et al. (2004), and the GrainGenes 2.0 database^2^.

The association mapping marker dataset was filtered using the following criteria: monomorphic markers and markers with >10% missing data or minor allele frequency (MAF) < 5% were omitted (Liu W.Z. et al., 2017). After applying these filtering criteria, 7,136 DArT-seq markers and 610 SSR markers were considered for the GWAS. Of the 7,136 DArT-seq markers that satisfied this criterion, 5,457 were positioned on the consensus genetic map. The polymorphic information content (PIC) and Shannon–Weaver diversity index (*H*′) were calculated for each DArT-seq and SSR marker using the formulae $PIC=1-\sum {\left(p_{\text{i}}\right)}^{2},$ and $H^{\prime }=-\sum_{\text{i}}^{\text{s}}p_{\text{i}}\ln p_{\text{i}},$ respectively, where *p*_i_ represents the proportion of the population carrying the *i*th allele (Botstein et al., 1980). A cluster analysis was performed using the neighbor-joining algorithm, and the shared-allele distance was used to determine the genetic structure of the accessions using PowerMarker v3.25 (Liu and Muse, 2005). The neighbor-joining tree was visualized using FigTree v1.4.3 (Muleta et al., 2017).

### Population Structure and Linkage Disequilibrium Analysis

A population structure analysis was performed using STRUCTURE v2.3.4 (Liu W.Z. et al., 2017). The dataset comprised 7,746 high-quality markers (MAF ≥ 5% and missing data ≤ 10%), including 7,136 DArT-seq and 610 SSR markers. Ten runs were performed with a *K*-value range of 1–10 using the admixture and correlated allele frequencies model with a burn-in of 100,000 iterations and Monte Carlo Markov Chain of 100,000 iterations (Liu W.Z. et al., 2017). The default settings were used for all other parameters. The optimal *K*-value was selected using the Δ*K* method (Evanno et al., 2005) (Supplementary Figure S1). Kinship among the 152 accessions was estimated using 7,746 markers with TASSEL v3.0 (Bradbury et al., 2007). *Q* and *K* were used in the mixed linear model as covariates to eliminate the moderately significant *P*-values that showed a breach of the expected distribution (Bulli et al., 2016). The LD between all pairs of markers was calculated using TASSEL v3.0 (Bradbury et al., 2007). The LD values across the known genetic distance for each chromosome of the 152 accessions were also estimated using TASSEL v3.0 with 5,457 DArT-seq markers (Evanno et al., 2005; Bradbury et al., 2007; Liu W.Z. et al., 2017). The mean *r*^2^ values over different genetic distances were also estimated for the whole genome. The LD decay plot was generated using *r*^2^ values and the genetic map distance between markers. The genetic distance at which the LD decay curve intersected with the critical *r*^2^ value was used as a threshold to determine the confidence intervals of significant QTL.

### Phenotyping and Phenotypic Data Analysis

Accessions were evaluated for APR against stripe rust by artificial inoculation with mixed *Pst* races in five field trials, which were performed in two locations of Sichuan Province. One trial was performed at Chongzhou (CZ; 30°33′N, 103°39′E) over three consecutive growing seasons (2015–2017) and the second trial was performed at Mianyang (MY; 31°23′N, 104°49′E) over two consecutive growing seasons (2015–2016). The different year-location combinations were defined as “environments.” The five environments were designated 15CZ, 16CZ, 17CZ, 15MY, and 16MY. In all of the test environments, all accessions were evaluated in three non-replicated rows. In total, 60 seeds of each accession were sown by hand in three rows with the 0.1 m inter-plant spacing in beds 2.0 m long, and the 0.3 m inter-row spacing (approximately 20 plants per row). Seeds of the susceptible cultivar ‘SY95-71,’ which is a Sichuan winter-wheat line susceptible to almost all Chinese *Pst* races, were sown every 20th row. Seeds of an additional susceptible cultivar, ‘Taichung 29,’ which is a Chinese commercial cultivar susceptible to almost all Chinese *Pst* races, were sown as spreader rows around each plot to ensure sufficient and homogenous distribution of *Pst* across the trials. The susceptible and spreader rows were inoculated approximately 1 month after planting with urediniospores of seven uniformly mixed *Pst* isolates prevalent in China (CYR 32, CYR 33, CYR 34, Shuiyuan 4, Shuiyuan 5, Shuiyuan 7, and Guinong 22–14). The aims of inoculating these *Pst* isolates in a mixture in the field were to screen for wheat accessions that exhibited broad-spectrum resistance, and to distinguish accessions that exhibited such resistance to stripe rust at the adult stage.

Stripe rust disease severity (DS), which was recorded as the percentage leaf area showing disease symptoms, was evaluated three times between the early and late dough stages. The first evaluation was performed when ‘SY95-71’ and ‘Taichung 29’ displayed DS values of at least 80%, and was followed by two additional evaluations at 7 days intervals. Resistance to stripe rust was measured using the “Rules for monitoring and forecasting wheat stripe rust (*Puccinia striiformis* West.)” (National Standard of the People’s Republic of China, GB/T 15795-2011). Infection type (IT) was visually scored on a 0–4 scale described by Bariana and McIntosh (1993) as follows: “0” = immune (no visible uredia); 0; = near immune (necrotic or chlorotic flecks without sporulation); 1 = highly resistant (small uredia with necrosis); 2 = moderately resistant (small to medium uredia with necrosis and chlorosis); 3 = moderately susceptible (medium-sized uredia with chlorosis); and 4 = highly susceptible (large uredia without chlorosis). Accessions with IT 0–2 were classified as resistant and those with IT 3–4 as susceptible.

Descriptive statistics and analysis of variance of stripe rust IT and DS data from the field experiments were performed using QTL IciMapping v4.1 (Meng et al., 2015; Yuan et al., 2018). Broad-sense heritability (*H*^2^) estimates were calculated for each environment as: *H*^2^ = δ_g_^2^/(δ_g_^2^ + δ_e_^2^), where δ_g_ and δ_e_ are estimates of genetic and environmental variances, respectively (Lu et al., 2016). To eliminate the environmental impact on stripe rust responses, the best linear unbiased prediction (BLUP) values (Piepho et al., 2008) were calculated using a mixed model procedure (PROC MIXED) with SAS v8.1 (SAS Institute Inc., Cary, NC, United States). Pearson’s correlation coefficients among environments were calculated to evaluate the consistency levels of stripe rust IT and DS values across the environments.

### GWAS for Stripe Rust

To identify loci associated with responses to stripe rust, a GWAS was performed using 7,746 high-quality markers, including 7,136 DArT-seq markers and 610 SSR markers, phenotypic data (IT and DS) from the five environments, and the BLUP values. The MTAs were identified using the mixed linear model, which incorporated the coefficients *Q* and *K* used in the adult-plant stage estimates of IT and DS with TASSEL v3.0 (Yu et al., 2006; Chen et al., 2017). The loci with significant MTAs had a -log_10_(*P*) threshold of 3. The DArT-seq and SSR markers were combined into a single putative QTL if they resided within a confidence interval of ±1.11 cM based on the standard critical threshold *r*^2^ = 0.3, in accordance with the method of Maccaferri et al. (2015b). We also compared the locations of significant QTL in the GWAS with those of previously reported *Yr* genes, including 80 formally named *Yr* genes (*Yr1–80*) and 67 temporarily designated *Yr* genes, and 332 mapped QTL were projected onto the integrated map that included DArT, SSR, and SNP markers using BioMercator v4.2 (Chen et al., 2017; Cheng et al., 2019). Comparison of DArT-seq marker positions was also carried out using the Wheat consensus genetic map v4.0^3^ and IWGSC RefSeq v1.0^4^ with BLAST+ v2.7.1 (Camacho et al., 2009).

## Results

### Genetic Diversity and Population Structure

In total, we identified 7,746 polymorphic markers (MAF ≥ 5% and missing data ≤ 10%) among the 152 wheat accessions. These markers were unevenly distributed among the three subgenomes and chromosomes of wheat. Of the polymorphic markers, 2,523, 3,506, and 1,717 markers were mapped to the A, B, and D subgenomes, respectively. Chromosome 3B contained the greatest number of markers (808), whereas chromosome 4D contained the fewest number of markers (97) (Supplementary Figure S2 and Supplementary Table S2). The MAF, gene diversity, and PIC indices were used to evaluate the extent of genetic variation among the 152 accessions. The three genetic diversity indices exhibited consistent trends that showed the population of wheat landraces contained high genetic diversity (Supplementary Figure S3).

The data for the three genetic diversity indices are presented in Supplementary Table S2. Genome-specific analyses of Nei’s (1973) genetic distance were significantly consistent among these indices. Chromosome 6A showed the greatest MAF, gene diversity, and PIC values of 0.267, 0.351, and 0.280, respectively, whereas chromosome 4A showed the lowest values of 0.196, 0.281, and 0.233, respectively. Among the three subgenomes, subgenome D showed the greatest MAF, gene diversity, and PIC values (0.232, 0.321, and 0.261, respectively), whereas subgenome A exhibited the lowest values (0.227, 0.313, and 0.255, respectively). The genome-wide means of the three indices for the 7,746 polymorphic markers were 0.230, 0.318, and 0.259, respectively (Supplementary Table S2 and Supplementary Figure S3).

**FIGURE 1:**
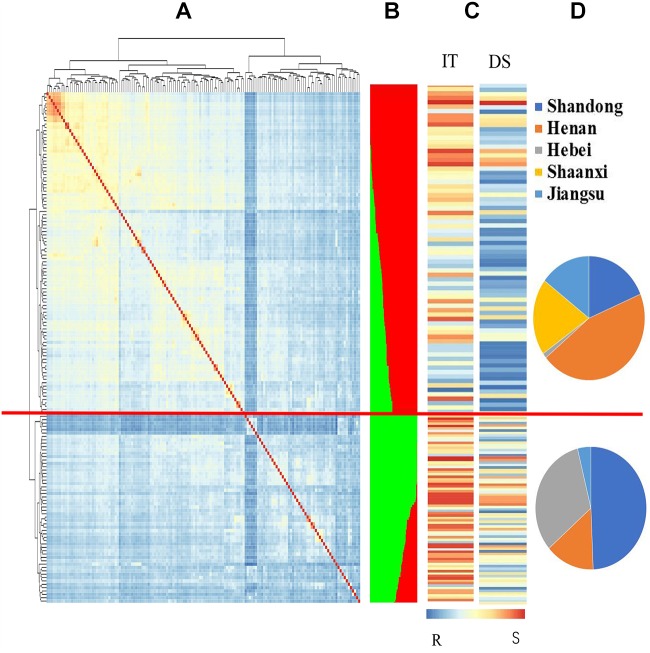
Relationship of population structure and genetic relatedness analysis of 152 Chinese Yellow and Huai River Valleys Wheat Zone landraces between population sub-clustering and stripe rust resistance. **(A)** Estimated the distance of hierarchical clustering for the accessions using Fast Ward grouping algorithm and heat map showing the kinship and phylogenetic relations. The same phylogeny was shown on the left and above the heatmap. Orange represents high kinship relations while blue colors shows continuous weaker relations. **(B)** Population structure summary plot (*k* = 2) of membership coefficients using STRUCTURE v.2.3.4. Each individual accession partitioned into colored segments, with the area of each segment representing the proportion. Two given sub-populations are represented: red Gp1; green Gp2. The red horizontal lines separated the 152 Chinese Yellow and Huai River Valleys Wheat Zone landraces into two subgroups according to structure membership coefficients. **(C)** The bar chart displayed IT and DS blue to white to red lines indicate reactions to *Pst* changed from resistance to intermediate to susceptibility to stripe rust. **(D)** Frequency of population structure subgroups in geographical regions of origin. From the top to the bottom, they are subgroups 1 and 2.

A neighbor-joining phylogenetic analysis based on shared allele distances showed that the 152 landraces exhibited a high degree of genetic relatedness (Supplementary Figure S4). Based on the greatest Δ*K* value using the 7,746 polymorphic markers, the 152 accessions were divided into two subgroups, Gp1 and Gp2 (Supplementary Table S1 and Figure 1B). Subgroup Gp1 contained 75 accessions, which were predominantly from Henan, Shaanxi, and Jiangsu Province. Subgroup Gp2 contained 77 accessions, which were predominantly from Shandong and Hebei Provinces. The geographical distribution of the subgroups is shown in Figure 1D. Based on the heat map for the IT and DS values of the 152 accessions, highly resistant accessions were clustered in Gp1, and the majority of the accessions in Gp1 were characterized by low IT and DS values (Supplementary Table S1 and Figure 1C, 2B,D). The mean IT values in the five environments for the five provinces ranged from 1.41 to 3.31, and the mean DS values ranged from 15.99 to 56.45%. The lowest IT and DS values were observed for Shaanxi Province (0.67–2.27 and 5.07–27.33%, respectively), whereas the greatest values were observed for Hebei Province (3.00–3.15 and 26.63–75.96%, respectively) (Table 1). The Fast Ward distance-based hierarchical clustering method, which explains the genetic structure of the population, revealed similar genetic variation among the accessions (Figure 1A). Kinship coefficients for the 152 accessions calculated with TASSEL v3.0 using the 7,746 polymorphic markers ranged from 0 to 1, with an average of 0.48. The kinship coefficients in Gp1 ranged from 0.52 to 1, with an average of 0.80, and those in Gp2 ranged from 0.51 to 1, with an average of 0.84.

**FIGURE 2:**
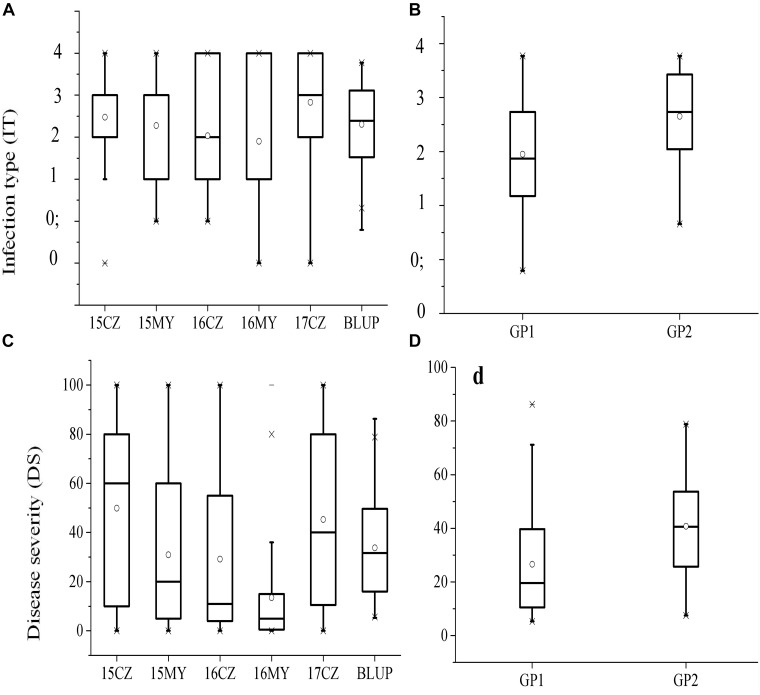
Box plot for distributions of IT **(A)** and DS **(C)** evaluated at the adult-plant stage in five environments; Mean, median, and range of IT **(B)** and DS **(D)** variation in each of the two subgroups at the adult-plant stage. Solid horizontal lines show medians. The circle signs the mean, the top and bottom box edges show the 25th to 75th percentiles of the total data, and the outer outliers.

**Table 1:** Analysis of stripe rust adult-plant resistance in five environments in five provinces.

Trait	Environment	Shandong	Henan	Hebei	Shaanxi	Jiangsu
IT	15CZ	2.90	1.80	3.46	1.53	2.21
	15MY	2.12	2.25	3.00	1.47	2.50
	16CZ	2.25	1.42	3.38	1.13	1.64
	16MY	1.62	1.89	3.15	0.67	2.00
	17CZ	2.88	2.60	3.54	2.27	2.64
	Mean	2.35	1.99	**3.31**	**1.41**	2.20
DS	15CZ	59.33	32.16	75.96	27.33	46.43
	15MY	28.94	29.09	50.58	11.67	28.21
	16CZ	24.99	24.29	57.50	10.30	28.07
	16MY	9.97	11.84	26.63	5.07	16.78
	17CZ	47.81	35.16	71.58	25.60	40.86
	Mean	34.21	26.51	**56.45**	**15.99**	32.07


*The mean values of IT and DS at the lowest and the highest were labeled in black bold*.

Across the 152 accessions, the genome-wide LD generally declined with genetic distance (cM). Pairwise DArT-seq markers showed a significant LD (*P* < 0.001), which was illustrated by the scatter of pairwise LD *r*^2^ values (Supplementary Figure S5). The baseline intersected with the smoothing spline curve at 1.11 cM based on the standard critical *r*^2^ = 0.3, which was used to estimate the QTL coverage regions with inter-marker genetic distance confidence intervals of ±1.11 cM from the peak of the significant associations.

### Phenotypic Assessment and *H*^2^ Estimation

The responses of the 152 wheat landraces to *Pst* were assessed in the five environments (3 years at CZ, and 2 years at MY). The phenotypic data used for the GWAS comprised the IT and DS values. Based on the IT data, two accessions (1.32%) were highly resistant (R, IT 0–1) to the mixed races of *Pst* across all environments at the adult-plant stage, whereas 17 accessions (11.18%) were highly susceptible (S, IT = 4). Based on the mean DS values, broad variation was exhibited among the 152 accessions in each environment, ranging from 8 to 57%. In total, 11.84% (mean DS < 20%) of accessions were highly resistant and 32.24% were highly susceptible (mean DS > 80%) to the mixed races of *Pst* across all environments at the adult-plant stage (Supplementary Table S1). The means for IT and DS ranged from 1.90 to 2.83 and 13.52 to 49.90%, respectively, within the environments (Figure 2A,C). Individual subpopulations showed different degrees of stripe rust resistance, with Gp1 showing the lowest mean BLUP values for IT (1.95) and DS (26.60%) (Figure 2B,D), which indicated the influence of APR genes. The means for IT and DS for the accessions originating from Shaanxi Province were greater than those from Hebei Province (Table 1). The phenotypic variation of IT and DS across the five environments was validated by phenotypic distributions based on BLUP values. In general, we identified 19 accessions with stable high-level resistance to stripe rust across all environments at the adult-plant stage, with low IT (0–2), DS (<20%), and BLUP (<1.20 for IT and <10.00 for DS) values (Supplementary Table S3). These accessions are promising sources of stripe rust resistance to exploit in breeding programs. Analysis of variance revealed statistically significant (*P* < 0.01) differences among the accessions in both the individual locations and across the locations in the five environments. The *H*^2^ values for stripe rust IT and DS, calculated across the five environments, were 81 and 86%, respectively, and collectively ranged from 77 to 86% (Table 2). The relatively high *H*^2^ estimates indicated environmental variation was limited compared with phenotypic variation across the five environments. The Pearson’s correlation coefficients for IT and DS responses to stripe rust ranged from 0.388 to 0.687 (Table 3). The Pearson’s correlation coefficients for stripe rust IT and DS among the multiple locations over multiple growing years averaged 0.574 and 0.541, respectively. Average correlations between years within locations were 0.571 and 0.632 for IT, and 0.493 and 0.611 for DS at CZ and MY, respectively. The correlation coefficients between IT and DS within the same environment ranged from 0.393 to 0.893 (Table 3).

**Table 2:** Estimates of variance components and heritability of IT and DS of stripe rust at adult-plant stage for the 152 Chinese Yellow and Huai River Valleys Wheat Zone landraces in five environments.

Parameter	Chongzhou		Mianyang		Across environments	
	IT (0–4)	DS (%)	IT (0–4)	DS (%)	IT (0–4)	DS (%)
Minimum	0	0	0	0	0	0
Maximum	4	100	4	100	4	100
Mean	2.45	41.46	2.09	22.22	2.30	33.76
*σ^2^_G_*	1.13**	745.86**	1.39**	413.08**	1.14**	536.22**
*σ^2^_E_*	–	119.40**	–	–	0.06**	211.66**
*σ^2^_G×E_*	–	465.34**	–	–	0.24**	390.54**
*σ^2^_e_*	0.72**	2.24**	0.73**	2.44**	0.71**	1.78**
*H^2^*	0.83	0.81	0.79	0.77	0.81	0.86


*σ^*2*^_*G*_, estimate of genotypic variance; σ^*2*^_*E*_, estimate of environmental variance; σ^*2*^_*G*__×__*E*_, estimate of genotype × environment variance; σ^*2*^_*e*_, estimate of residual variance; H^*2*^, broad-sense heritability; IT, infection type; DS, disease severity; –, not significant; ^∗^P < 0.05, ^∗∗^P < 0.01.*

**Table 3:** Correlation coefficients for IT and DS response to APR of 152 Chinese Yellow and Huai River Valleys Wheat Zone landraces in five environments.

IT vs. IT^a^	15MY	15CZ	16MY	16CZ	17CZ
15MY	1	0.591^∗∗^	0.632^∗∗^	0.634^∗∗^	0.585^∗∗^
15CZ		1	0.416^∗∗^	0.687^∗∗^	0.505^∗∗^
16MY			1	0.518^∗∗^	0.648^∗∗^
16CZ				1	0.520^∗∗^
17CZ					1
DS vs. DS^b^	15MY	15CZ	16MY	16CZ	17CZ
15MY	1	0.611^∗∗^	0.611^∗∗^	0.484^∗∗^	0.549^∗∗^
15CZ		1	0.608^∗∗^	0.681^∗∗^	0.411^∗∗^
16MY			1	0.569^∗∗^	0.493^∗∗^
16CZ				1	0.388^∗∗^
17CZ					1
IT vs. DS^c^	15MY	15CZ	16MY	16CZ	17CZ
15MY	**0.893^∗^**^∗d^	0.574^∗∗^	0.669^∗∗^	0.620^∗∗^	0.553^∗∗^
15CZ	0.588^∗∗^	**0.781**^∗∗^	0.471^∗∗^	0.718^∗∗^	0.456^∗∗^
16MY	0.564^∗∗^	0.531^∗∗^	**0.808^∗^**^∗^	0.621^∗∗^	0.530^∗∗^
16CZ	0.441^∗∗^	0.491^∗∗^	0.412^∗∗^	**0.693**^∗∗^	0.393^∗∗^
17CZ	0.492^∗∗^	0.404^∗∗^	0.607^∗∗^	0.488^∗∗^	**0.826^∗^**^∗^


*^*a*^Comparisons between the infection type of different environments. 15MY = 2015 Manyang; 15CZ = 2015 Chongzhou; 16MY = 2016 Manyang; 16CZ = 2016 Chongzhou; 17CZ = 2017 Chongzhou; ^*b*^Comparisons between the disease severity of different environments; ^*c*^Comparison between the IT and DS of different environments; ^*d*^Correlation coefficients between IT and DS of the same environment were labeled in bold; The P-values of all the correlation coefficients in the table use QTL IciMapping (P < 0.01).*

### Candidate Genes Associated With *Pst*

Using 7,746 polymorphic markers, a GWAS was performed for stripe rust IT and DS after exposure to mixed *Pst* isolates within the five environments and for BLUP values at the adult-plant stage based on the mixed linear model. A total of 51 markers within 40 distinct QTL located on all 16 chromosomes were determined to be significantly associated (*P* < 0.001) with APR (Table 4). Unique QTL were located on chromosomes 2D, 3A, 4A, and 6D, and more than one QTL on the other chromosomes. The phenotypic variance explained by each of these MTAs ranged from 7.44 to 17.70%. Detailed information on the 40 putative resistance QTL is presented in Table 4.

**Table 4:** Association of the 7746 markers at adult-plant stage of putative QTL to previously reported *Yr* genes and QTL.

Trial	QTL	Environment	Marker	PVE	Chr^a^	Position (cM)^a^	Marker interval	MapName	References
IT	*QIT.sicau-2A.1*	17CZ	*993186*	7.69%	2A	83.25	*Xwmc407*-*Xwmc170*	*QYrtm.pau-2A*	Chhuneja et al., 2008
	*QIT.sicau-2A.2*	15CZ	*1115258*	11.08%	2A	123.66	*Xgwm382a-Xgwm359*	*QYr.inra-2AL*	Mallard et al., 2005
	*QIT.sicau-3B*	16MY	*Xgwm389*	10.29%	3B	–	*Xgwm389-Xgwm493*	*QTL-3BS*	Suenaga et al., 2003
	*QIT.sicau-4A*	15MY	*1161461*	10.74%	4A	24.08	*IWA1940-IWA1941*	*Qyr.wpg-4A.1*	Naruoka et al., 2015
	*QIT.sicau-4B*	15MY, BLUP-IT	*2292362*	9.64–12.48%	4B	32.63	*–*	*–*	–
		15MY	*3022885*	10.61%	4B	32.63			
	*QIT.sicau-5B.1*	17CZ	*1266228*	9.10%	5B	42.96	*IWA6867-IWA6383*	*QYrdr.wgp-5BL.2*	Hou et al., 2015
	*QIT.sicau-5B.2*	15CZ	*1301734*	7.54%	5B	74.48	*wPt-3661-wPt-2707*	*QYr.sun-5B*	Bariana et al., 2010
	*QIT.sicau-7A*	16MY	*1309937*	9.98%	7A	75.85	*wPt-4877-wPt-4345*	*QYr.sun-7A*	Zwart et al., 2010
	*QIT.sicau-7D*	15MY	*Xcfd14*	7.76%	7D	–	*–*	*–*	–
DS	*QDS.sicau-1B*	15CZ	*3533134*	9.79%	1B	257.66	*wPt-1770 - wPt-9028*	*QYr.cim-1BL*	Lan et al., 2014
	*QDS.sicau-1D*	16MY	*1080456*	9.92%	1D	11.06	*–*	*–*	–
	*QDS.sicau-2A*	15MY	*3533777*	10.04%	2A	8.26	*–*	*–*	–
	*QDS.sicau-2B.1*	15MY	*5332256*	9.66%	2B	41.54	*wPt-9668–Xgwm429*	*QYrid.ui-2B.1*	Chen et al., 2012
	*QDS.sicau-2B.2*	16CZ,16MY	*Xbarc55*	8.80–12.62%	2B	–	*Xbarc13-Xbarc230*	*QYr.caas-2BS*	Lan et al., 2010
	*QDS.sicau-2D*	17CZ, BLUP-DS	*1115820*	10.20–17.70%	2D	130.86	*–*	*–*	–
	*QDS.sicau-4B.1*	15MY	*1089133*	10.08%	4B	45.05	wPt-8543 *-Xwmc238*	*QYr.sun-4B*	Zwart et al., 2010
	*QDS.sicau-4B.2*	16MY	*1218468*	10.67%	4B	78.18	*–*	*–*	–
	*QDS.sicau-5B*	15CZ	*Xgwm234*	9.53–10.70%	5B	–	*–*	*–*	–
	*QDS.sicau-6A.1*	16CZ	*1127951*	11.13%	6A	42.99	*–*	*–*	–
	*QDS.sicau-6A.2*	16MY	*1244540*	11.55%	6A	48.23	*–*	*–*	–
	*QDS.sicau-6A.3*	16MY, BLUP-DS	*3955268*	8.00–9.29%	6A	87.60	*–*	*–*	–
	*QDS.sicau-6A.4*	16MY	*3533288*	10.87%	6A	98.64	*–*	*–*	–
		16MY	*3385073*	10.13%	6A	99.20			
DS	*QDS.sicau-6B.1*	16MY	*1268178*	11.74%	6B	11.00	*IWA3297-IWA6436*	*QYrdr.wgp-6BL.1*	Hou et al., 2015
	*QDS.sicau-6B.2*	15MY	*1159379*	11.36%	6B	54.11	*–*	*–*	–
	*QDS.sicau-6D*	16CZ	*1209024*	10.29%	6D	0.57	*–*	*–*	–
	*QDS.sicau-7A.1*	15MY	*1100222*	9.82%	7A	6.23	*–*	*–*	–
		16MY, BLUP-DS	*1250999*	9.48–1	7A	41.89	*–*	*–*	–
	*QDS.sicau-7A.2*			0.60%			–	–	–
	*QDS.sicau-7A.3*	16MY	*1708004*	11.94%	7A	57.78			
	*QDS.sicau-7B.1*	16CZ	*1081577*	10.19%	7B	74.02	*wPt-9467-wPt-3723*	*QYr.sun-7B*	Bariana et al., 2010
	*QDS.sicau-7B.2*	15CZ	*Xwmc335*	9.15%	7B	–	*Xbarc72-Xwmc335*	*QyrPI182103.wgp-7BL*	Feng et al., 2018
	*QDS.sicau-7B.3*	15CZ	*Xwmc581*	8.99%	7B	–	*–*	*–*	–
	*QDS.sicau-7D.1*	16MY	*4910049*	9.89%	7D	52.39	*Xbcd1438-Xwg834*	*Yr18*–7DS	Singh et al., 2000
	*QDS.sicau-7D.2*	15CZ	*2242944*	9.97%	7D	154.81	*–*	*–*	–
	*QDS.sicau-7D.3*	15CZ	*Xcfd68*	11.94%	7D	–	*–*	*–*	–
IT, DS	*QYr.sicau-1B*	15MY	*1287759*	10.30%	1B	43.70	*Xgwm374- Xbarc181*	*QYr.cau-1BS*	Quan et al., 2013
		16CZ	*3938149*	8.39%	1B	48.80			
		15MY	*Xgwm374*	11.95%	1B	–			
		17CZ	*Xwmc611*	11.13%	1B	–			Hou et al., 2015
	*QYr.sicau-1D*	17CZ, BLUP-IT	*1022670*	8.8–10.63%	1D	46.83	*Xwmc432-Xgdm33.2*	*QYrdr.wgp-1DS.1*	Hou et al., 2015
		15CZ	*Xgwm337*	7.44%	1D	–			
	*QYr.sicau-2B*	16MY	*4394902*	13.87%	2B	81.05	*wPt-8460–wPt-3755*	*QYr.caas-2BL*	Ren et al., 2012
		16MY	*2278639*	10.40%	2B	90.58			
	*QYr.sicau-3A*	16MY	*1150091*	10.10%	3A	4.62	*wPt-6422-wPt-7890*	NA	Rosewarne et al., 2012
		17CZ	*1150427*	10.31%	3A	44.75			
		15MY	*1105026*	12.11%	3A	46.82			
		15MY	*Xcfd79*	10.84%	3A	–			
	*QYr.sicau-3B*	15CZ	*1289226*	10.17–11.73%	3B	52.58	*wPt-0267- wPt-10546*	*QYrpi.vt-3BL*	Christopher et al., 2013
								
IT, DS	*QYr.sicau-7D*	16MY	*4440148*	9.94%	7D	12.25	*–*	*–*	–
		15CZ,15MY, BLUP-IT	*3937237*	10.11–12.82%	7D	12.41			


*^*a*^Wheat consensus map version 4.0 (https:/www.diversityarrays.com/technology-and-resources/genetic-maps/); PVE, phenotypic variation explained; Chr, chromosome; “–”, not; NA, not applicable.*

Of the 40 QTL, nine QTL detected on seven chromosomes were associated with IT, explaining 7.54–12.48% of the phenotypic variation, and 25 QTL detected on chromosomes 1A, 3A, 4B, 6B, and 6D were associated with DS, explaining 8.00–12.62% of the phenotypic variation. In total, six of the 40 QTL were identified as associated with both IT and DS.

Twenty potentially novel QTL or *Yr* genes were associated with adult-plant responses, which were located on 11 chromosomes and explained 2.63–17.70% of the phenotypic variance (Table 5). In particular, *QYr.sicau-7D* was significantly associated with both IT and DS. *QDS.sicau-2A*, *QIT.sicau-4B*, *QDS.sicau-4B.2*, *QDS.sicau-6A.3*, and *QYr.sicau-7D* were significantly associated with adult-plant responses in four or more environments as well as with BLUPs, and explained 2.69–12.82% of the phenotypic variation. All of these novel loci are strong candidates to aid in development of cultivars with increased resistance to stripe rust at the adult-plant stage.

**Table 5:** Potentially novel QTL or *Yr* genes associated with stripe rust resistance to five environments and BLUPs at the adult-plant stage.

QTL	Marker	Chr	Trait	PVE	Marker-trait association significant level					
					15CZ	15MY	16CZ	16MY	17CZ	BLUP
*QDS.sicau-1D*	*1080456*	1D	DS	5.24–9.92%	NA	^∗∗^	NA	^∗∗∗^	NA	^∗^
***QDS.sicau-2A***	*3533777*	2A	DS	4.22–10.04%	^∗^	^∗∗∗^	^∗^	NA	^∗^	^∗∗^
*QDS.sicau-2D*	*1115820*	2D	DS	10.20–17.70%	NA	NA	NA	NA	^∗∗∗^	^∗∗∗^
***QIT.sicau-4B***	*2292362*	4B	IT	3.52–12.48%	^∗^	^∗∗∗^	^∗^	NA	^∗^	^∗∗^
	*3022885*	4B	IT	4.08–10.61%	^∗∗^	^∗∗∗^	^∗^	^∗^	NA	^∗∗^
***QDS.sicau-4B.2***	*1218468*	4B	DS	4.65–10.67%	NA	^∗^	^∗^	^∗∗∗^	^∗^	^∗∗^
*QDS.sicau-5B*	*Xgwm234*	5B	DS	9.53–10.70%	^∗∗∗^	NA	NA	NA	NA	NA
*QDS.sicau-6A.1*	*1127951*	6A	DS	11.13%	NA	NA	^∗∗∗^	NA	NA	NA
*QDS.sicau-6A.2*	*1244540*	6A	DS	4.26–11.55%	NA	^∗^	NA	^∗∗∗^	NA	^∗^
***QDS.sicau-6A.3***	*3955268*	6A	DS	8.00–9.29%	^∗^	^∗∗^	NA	^∗∗∗^	^∗∗^	^∗∗∗^
*QDS.sicau-6A.4*	*3533288*	6A	DS	10.87%	NA	NA	NA	^∗∗∗^	NA	NA
	*3385073*	6A	DS	10.13%	NA	NA	NA	^∗∗∗^	NA	NA
*QDS.sicau-6B.2*	*1159379*	6B	DS	11.36%	NA	^∗∗∗^	NA	^∗^	^∗^	^∗∗^
*QDS.sicau-6D*	*1209024*	6D	DS	10.29%	NA	NA	^∗∗∗^	NA	NA	NA
*QDS.sicau-7A.1*	*1100222*	7A	DS	9.82%	NA	^∗∗∗^	^∗^	NA	NA	^∗^
*QDS.sicau-7A.2*	*1250999*	7A	DS	9.48–10.60%	^∗^	^∗^	^∗∗^	^∗∗∗^	NA	^∗∗∗^
*QDS.sicau-7A.3*	*1708004*	7A	DS	11.94%	NA	NA	NA	^∗∗∗^	NA	NA
*QDS.sicau-7B.3*	*Xwmc581*	7B	DS	2.63–8.99%	^∗∗∗^	NA	^∗^	NA	NA	NA
*QIT.sicau-7D*	*Xcfd14*	7D	IT	3.31–7.76%	NA	^∗∗∗^	NA	^∗^	NA	^∗^
*QDS.sicau-7D.2*	*2242944*	7D	DS	4.65–9.97%	^∗∗∗^	^∗^	NA	^∗∗^	NA	^∗^
*QDS.sicau-7D.3*	*Xcfd68*	7D	DS	4.54–11.94%	^∗∗∗^	NA	^∗^	^∗∗^	NA	^∗^
***QYr.sicau-7D***	*4440148*	7D	IT	9.94%	NA	NA	NA	^∗∗∗^	NA	NA
	*3937237*	7D	DS	4.27–12.82%	^∗∗∗^	^∗∗∗^	^∗^	^∗∗^	NA	^∗∗∗^


*^∗^P < 0.05, ^∗∗^P < 0.005, ^∗∗∗^P < 0.001; NA, not applicable; Putative QTL that have significant association with adult-plant response in four or more environments as well as BLUPs, which are given in bold.*

## Discussion

### LD Decay and Population Structure

Population structure is an important factor that influences LD (Flint-Garcia et al., 2003). Assessment of population structure is extremely important before conducting a GWAS to avoid spurious associations (Yu et al., 2006). In the present study, STRUCTURE analysis divided the 152 wheat accessions into two subgroups on the basis of genotype data. The *Q* and *K* method was used in the GWAS analysis, and some false negative MTAs were eliminated (Liu Y.X. et al., 2017).

### Response of Adult-Stage Wheat Landraces to Stripe Rust

In this study, we evaluated responses to stripe rust across five environments. The statistically significant (*P* < 0.001) differences observed in different environments were most likely the result of variation in environmental variables (temperature and rainfall) and the *Pst* race composition in each environment. Some accessions showed lower IT and DS values in one environment compared with those in a different environment. In this situation, BLUP values were obtained across locations and years, with genotypes considered as fixed effects in this model (Liu W.Z. et al., 2017; Zhang et al., 2018). Thus, to increase the reliability of the results, MTAs were considered relevant only when the parameters were significant in two or more environments, as well as in the multi-environment BLUP analysis.

Identification and mapping of stripe rust resistance genes have been conducted since the 1960s (Lupton and Macer, 1962). The majority of stripe rust resistance genes have been identified in common wheat (Chen, 2013). Chinese landraces, such as ‘Pingyuan 50,’ which may possess potentially useful loci for race-specific and race-non-specific resistance, have been investigated (Lan et al., 2010). In the current study, MTAs were not associated with multiple environments when applying a stringent significance level in each environment. When applying *P* < 0.001, only *QYr.sicau-7D*, located in the QTL region between *4440148* and *3937237*, was highly significantly associated with IT and DS in three environments as well as with BLUPs at the adult-plant stage (Table 4). When applying the less stringent significance criterion of *P* < 0.005, five additional loci (*QDS.sicau-6A.3*, *QDS.sicau-7A.2*, *QYr.sicau-1D*, *QYr.sicau-4B.1*, and *QYr.sicau-4B.2*) were significantly associated with IT and/or DS in two or more environments as well as with BLUPs at the adult-plant stage. Three of the five loci (*QDS.sicau-6A.3*, *QYr.sicau-4B.1*, and *QYr.sicau-4B.2*) were potentially novel APR loci. The QTL *QYr.sicau-4B.1* and *QYr.sicau-4B.2* were identical to *QIT.sicau-4B* and *QDS.sicau-4B.2*, respectively, and were significantly associated with IT and DS (Table 5 and Supplementary Table S4). Seven additional loci (*QIT.sicau-2B.1*, *QDS.sicau-3B*, *QIT.sicau-5B*, *QDS.sicau-5D*, *QIT.sicau-6A*, *QDS.sicau-6D.1*, and *QDS.sicau-7A*) were significantly associated (*P* < 0.05) with IT and/or DS in four or more environments as well as with BLUPs at the adult-plant stage (Table 4 and Supplementary Table S4). The other loci that showed significant associations with IT and DS in single environments should be treated with caution because they were instable in different environments and susceptible to some *Pst* races.

### Multigenic and Pleiotropic Effects Revealed by GWAS

Multigenic effects were observed in the current study, and IT and DS were significantly associated with multiple markers. Six QTL (*QYr.sicau-1B*, *QYr.sicau-1D*, *QYr.sicau-2B*, *QYr.sicau-3A*, *QYr.sicau-3B*, and *QYr.sicau-7D*) were highly significantly associated (*P* < 0.001) with IT and DS. For example, *QYr.sicau-1B*, *QYr.sicau-1D*, *QYr.sicau-2B*, *QYr.sicau-3A*, and *QYr.sicau-7D* were associated with two or more markers, identified as *Xgwm374*, *Xwmc611*, *1287759*, and *3938149*; *1022670* and *Xgwm337*; *4394902* and *2278639*; *1150091*, *1150427*, *1105026*, and *Xcfd79*; and *4440148* and *3937237*, respectively (Table 4). Of 20 potentially novel QTL, five (*QDS.sicau-2A*, *QIT.sicau-4B*, *QDS.sicau-4B.2*, *QDS.sicau-6A.3*, and *QYr.sicau-7D*) were significantly (*P* < 0.05) associated with IT and/or DS in four or more environments, as well as with BLUPs, at the adult-plant stage (Tables 4, 5). The phenotypic variation explained by these five loci ranged from 9.29 to 12.82%. Therefore, the five QTL may have large effects on APR, particularly *QIT.sicau-4B* and *QYr.sicau-7D*, which contained multiple markers. These five QTL were used to research the potential molecular functions of the significant markers and the putative QTL.

### Association of Significant Resistance Loci With Previously Published *Yr* Genes or QTL

Using the 152 accessions from the Yellow and Huai River Valleys, 40 putative QTL were detected that were significantly (*P* < 0.001) associated with APR to stripe rust caused by a mixture of prevalent *Pst* races. Of these QTL, 20 had been previously published. *QIT.sicau-5B.1*, *QYr.sicau-1B*, and *QYr.sicau-1D* were identified in winter wheat. The markers located near *QYrdr.wgp-5BL.2* were *IWA6383* and *IWA6867*, and *QIT.sicau-5B.1* was flanked by the QTL region in the same interval. *QYr.sicau-1B* was significantly associated with markers *1287759*, *3938149*, *Xgwm374*, and *Xwmc611*, which were previously published in winter wheat and located in an interval containing *Xgwm374* and *Xwmc611* (Quan et al., 2013; Hou et al., 2015). *QYrdr.wgp-1DS.1* was flanked by *Xgwm353*, *Xgdm33b*, *Xgwm337*, and *Xwmc432*. In the present study, *QYr.sicau-1D* was located close to this QTL region. Thus, these two QTL could be identical (Hou et al., 2015). *QIT.sicau-5B.2* and *QDS.sicau-6B.1* were consistently identified in the wheat cultivars ‘Janz’ and ‘Kukri,’ respectively (Bariana et al., 2010). The QTL *QYrtm.pau-2A* was previously mapped to a 3.6-cM interval between *Xwmc407* and *Xwmc170*, whereas *QIT.sicau-2A.1* from the present study was mapped proximal to this QTL (Bariana et al., 2010). The QTL *QIT.sicau-2A.2* was identified in the interval between *Xgwm382a* to *Xgwm359* (Mallard et al., 2005). *QIT.sicau-3B*, identified in bread wheat, is associated with *Xgwm389* on chromosome 3BS and is considered to affect *Yr30* (Suenaga et al., 2003). *QIT.sicau-4A*, also reported as *Qyr.wpg-4A.1*, is located in the interval between *IWA1940 to IWA1941* (Naruoka et al., 2015). *QIT.sicau-7A* is inherited from a synthetic hexaploid parent (CPI133872) and is located on the distal part of chromosome 7AS. *QDS.sicau-1B* reduced the stripe rust DS value. Closely linked markers suggest that the 1BL locus has pleiotropic and multigenic effects on the APR gene *Lr46/Yr29* (Lan et al., 2014). Stripe rust resistance QTL on chromosome 2B were previously reported (Ramburan et al., 2004; Guo et al., 2008; Carter et al., 2009). *QDS.sicau-2B.1* may be a major locus derived from IDO444 (Chen et al., 2012). The SSR marker *Xbarc55*, which is closely linked to *QDS.sicau-2B.2*, may be useful to improve wheat stripe rust resistance (Lan et al., 2010). Chromosome 4BL was identified as containing many QTL (Rosewarne et al., 2013) and was the most affected by segregation distortion. *QDS.sicau-4B.1*, also reported as *QYr.sun-4B*, is located in a 4.3-cM interval containing *wPt-8543* and *Xwmc238* (Zwart et al., 2010) but was not detected in the present GWAS analysis. Chromosomes 7B and 7D have been reported to be associated with stripe rust resistance responses (Pink and Law, 1985). A QTL region on chromosome 7B reduces pustule density, which is a rust disease component (Muhammad et al., 2005). *QDS.sicau-7B.1* is flanked by *wPt-9467* and *wPt-3723* in *QYr.sun-7B* (Bariana et al., 2010). *QDS.sicau-7B.2*, which is located close to marker *Xwmc335*, was identified in *QyrPI182103.wgp-7BL* as being linked to *Xbarc72* and flanked by the *Yr79* locus (Feng et al., 2018). *QDS.sicau-7D.1* was identified by the *Yr18* gene, and the region contributed by the cultivar ‘Opata 85’ was observed to reduce DS by almost half in all trials (Singh et al., 2000). *QYr.sicau-2B* is contributed by the common wheat cultivar ‘Naxos’ (Ren et al., 2012) in the marker interval *XwPt*-*8460* to *XwPt*-*3755*, which was significantly associated with IT and DS, and these resistant loci were tagged by *4394902* and *2278639*. The QTL *QYr.sicau-3A*, which was significantly associated with IT and DS, was flanked by *wPt-6422* and *wPt-7890* in an ‘Avocet’ × ‘Pastor’ wheat population (Rosewarne et al., 2012). Christopher et al. (2013) reported previously that *QYrpi.vt-3BL*, which is located between markers *wPt-0267* and *wPt-10546*, explained 10.17–11.73% of the phenotypic variation. On the basis of the genetic locations of *QYrpi.vt-3BL* and *QYr.sicau-3B* on chromosome 3BL in the present study, these two QTL might be closely linked or located in the same chromosomal region.

### Novel Stripe Rust Resistance Loci

**FIGURE 3:**
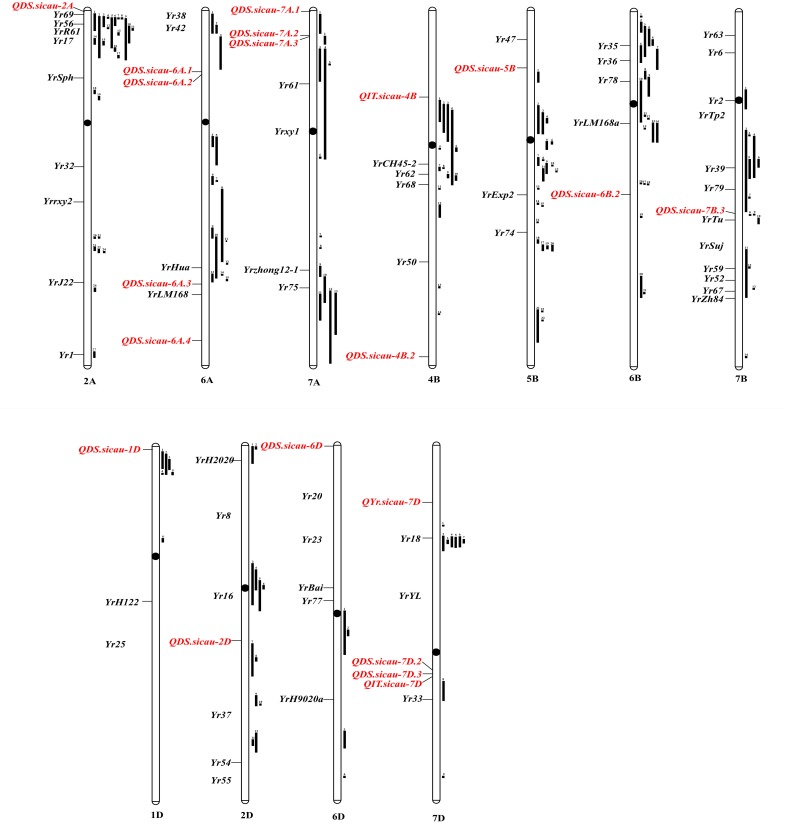
Chromosomal positions of loci associated with IT and/or DS to Pst identified in this study relative to positions of previously mapped QTL and *Yr* genes. The relative lengths of all chromosomes are standardized same. Loci identified in this study are highlighted in red. Previously mapped QTL (black bar) is on right side of the chromosomes and *Yr* genes (black) for stripe rust resistance is on left side of the chromosomes. All positions are approximations, and thus could be served as guidelines for future research. The relationships between loci markers identified in this study and the previously mapped QTL and *Yr* genes are described (Table 4 and Supplementary Table S5).

A total of 20 potentially novel QTL that are significantly (*P* < 0.001) associated with IT and/or DS were detected in the field at the adult-plant stage. The relative positions and details of the previously mapped QTL and *Yr* genes on the integrated map are shown in Figure 3 and Supplementary Table S5. These represent potentially novel resistance loci because no QTL or genes are reported in the same interval (Table 5). However, allelism tests are required to determine which represent alleles of previously mapped genes and which represent novel *Yr* genes.

The subgenome A contained eight QTL, which were located on chromosomes 2A, 6A, and 7A. Of these QTL, six explained 10.04–11.94% of the phenotypic variance. *QDS.sicau-2A* was identified on the distal 3.81% of the short arm of chromosome 2A and was linked to previously reported QTL (Maccaferri et al., 2015b). However, *3533777* was located in a different linkage region. Therefore, *QDS.sicau-2A* is a potentially novel QTL. There are no genetic maps available to compare relative distances; therefore, the identity of *QDS.sicau-2A* needs to be confirmed using an allelism test. *QDS.sicau-6A.1* and *QDS.sicau-6A.2* were assigned to the wheat chromosome 6AS. Currently, there are no genetic maps available to compare relative distances between both *1127951* and *1244540* and the flanking markers for these previously reported QTL. Therefore, more work is required to determine whether *QDS.sicau-6A.1* and *QDS.sicau-6A.2* are novel. *QDS.sicau-6A.4* was located on chromosome 6AL and assigned to the distal 15.97% of the long arm; this QTL may be a novel APR locus because there is no previously reported QTL in this region. *QDS.sicau-7A.2* and *QDS.sicau-7A.2* were assigned to the distal 5.22 and 7.31% of the short arm of wheat chromosome 7A, respectively. Many QTL have been previously reported on chromosome 7AS (Zwart et al., 2010; Rosewarne et al., 2012; Maccaferri et al., 2015a; Liu Y.X. et al., 2017), but not in this chromosomal region.

In subgenome B, one QTL was located on each of the chromosomes 5B, 6B, and 7B, and two QTL on chromosome 4B. Four out of five QTL explained 10.61–11.36% of the phenotypic variance. *QIT.sicau-4B* was located on chromosome 4BS, which harbors a number of previously reported QTL (Agenbag et al., 2012; Liu Y.X. et al., 2017), but these differ from *QIT.sicau-4B*. *QDS.sicau-4B.2* was assigned to the distal 3.98% of the long arm of chromosome 4B, which to the best of our knowledge does not overlap with the position of any known APR gene; therefore, this QTL may represent a novel resistance locus. *QDS.sicau-5B*, which was located on the short arm of chromosome 5B, may be a novel QTL because only *Yr47* and *QYr.uga-5B*, which are not located in this region, have been published (Hao et al., 2011). *QDS.sicau-6B.2* was located on 6BL, and the previously published QTL (William et al., 2006; Rosewarne et al., 2012; Liu Y.X. et al., 2017) differ from *QDS.sicau-6B.2*.

Seven QTL were located in subgenome D, with one QTL each located on chromosomes 1D, 2D, and 6D, and four QTL were located on chromosome 7D. The foremost of these QTL was *QYr.sicau-7D*, which was highly significantly associated with IT and DS in three environments as well as with BLUPs at the adult-plant stage. In addition, four out of seven QTL explained 10.20–17.70% of the phenotypic variance. *QYr.caas-2DL* and *Yr54* were previously published (Basnet et al., 2014), but differ from *QDS.sicau-2D* detected in the present study. Thus, *QDS.sicau-2D* is likely a novel stripe rust resistance locus. *QDS.sicau-6D*, which was assigned to the distal 0.01% of the short arm of chromosome 6D, represents a novel resistance locus because no race-specific genes have been published in this chromosomal region. *QDS.sicau-7D.3* was mapped in proximity to the centromere of chromosome 7DL. There are no reports of any significant associations with stripe rust responses in this chromosomal region (Boukhatem et al., 2002). The confidence interval of *QYr.sicau-7D* tagged by DArT markers *4440148* and *3937237* did not overlap with the position of a previously published *Yr* gene or QTL, and thus it is likely a novel stripe rust resistance locus.

## Author Contributions

LL carried out the experiments, analyzed the data, and drafted the manuscript. FY, CY, XY, YC, YqW, YW, JL, and MD performed the experiments. JW, QJ, WL, JM, and YmW analyzed the data. YL revised the manuscript. YZ participated in the design of the experiments. GC formulated the questions, designed and carried out the experiments, analyzed the data, and revised the manuscript. All authors read and approved the final version.

## Conflict of Interest Statement

The authors declare that the research was conducted in the absence of any commercial or financial relationships that could be construed as a potential conflict of interest.

## Footnotes

1 http://www.diversityarrays.com/

2 http://wheat.pw.usda.gov/GG2

3 https:/www.diversityarrays.com/technology-and-resources/genetic-maps/

4 http://www.wheatgenome.org/
