# Supplementary material for: Genome-Wide Association Study for Adult-Plant Resistance to Stripe Rust in Chinese Wheat Landraces (Triticum aestivum L.) From the Yellow and Huai River Valleys
Source: Front Plant Sci. 2019 May 16;10:596. doi: 10.3389/fpls.2019.00596 (PMC6532019; doi:10.3389/fpls.2019.00596)
Supplement: Supplementary file 6 [file Data_Sheet_1.docx]

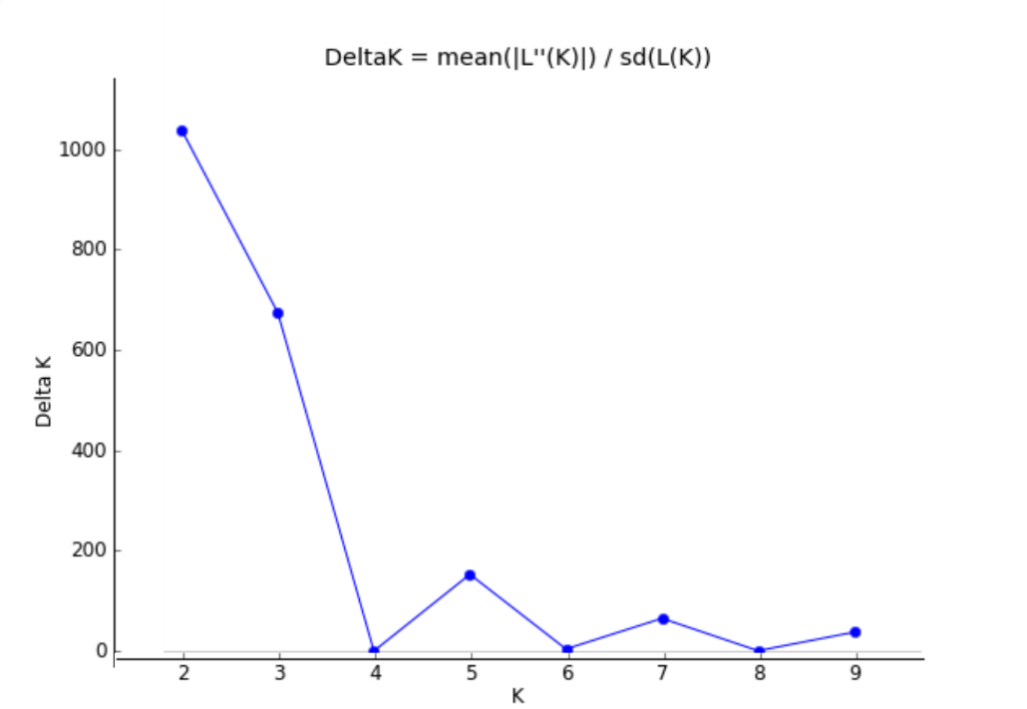


**Figure S1.** Analysis of the population structure of 152 wheat accessions, Delta *K* based on the rate of change of LnP(D) between successive *K*.
