# Supplementary figures and images for: Genome-Wide Association Study for Adult-Plant Resistance to Stripe Rust in Chinese Wheat Landraces (Triticum aestivum L.) From the Yellow and Huai River Valleys
Source: Front Plant Sci. 2019 May 16;10:596. doi: 10.3389/fpls.2019.00596 (PMC6532019; doi:10.3389/fpls.2019.00596)

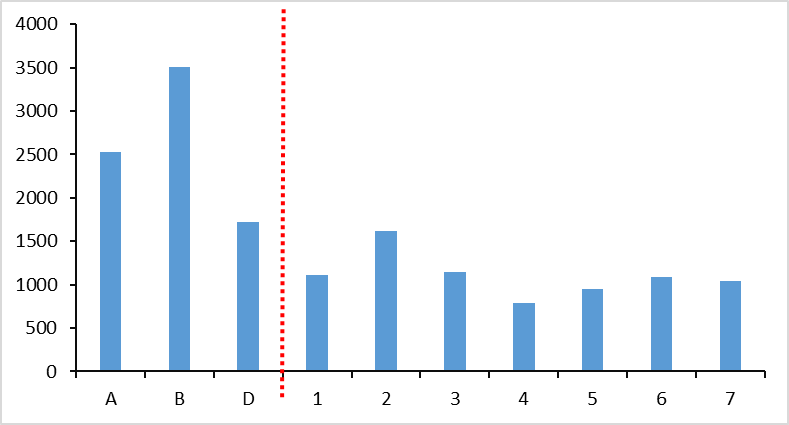


**Figure S2.** 7746 polymorphism markers distributions were obtained for the 152 accessions.

Supplement: Supplementary file 7 [file Data_Sheet_2.docx]
