# Supplementary material for: Genome-Wide Association Study for Adult-Plant Resistance to Stripe Rust in Chinese Wheat Landraces (Triticum aestivum L.) From the Yellow and Huai River Valleys
Source: Front Plant Sci. 2019 May 16;10:596. doi: 10.3389/fpls.2019.00596 (PMC6532019; doi:10.3389/fpls.2019.00596)
Supplement: Supplementary file 8 [file Data_Sheet_3.docx]

**a**

**b**

**Figure S3** Genome specific comparisons of molecular diversity in landrace accessions. Nei’s gene diversity, PIC values and MAF were used to compare the extent of genetic variation in landrace accessions.
