# Supplementary material for: Genome-Wide Association Study for Adult-Plant Resistance to Stripe Rust in Chinese Wheat Landraces (Triticum aestivum L.) From the Yellow and Huai River Valleys
Source: Front Plant Sci. 2019 May 16;10:596. doi: 10.3389/fpls.2019.00596 (PMC6532019; doi:10.3389/fpls.2019.00596)
Supplement: Supplementary file 9 [file Data_Sheet_4.docx]

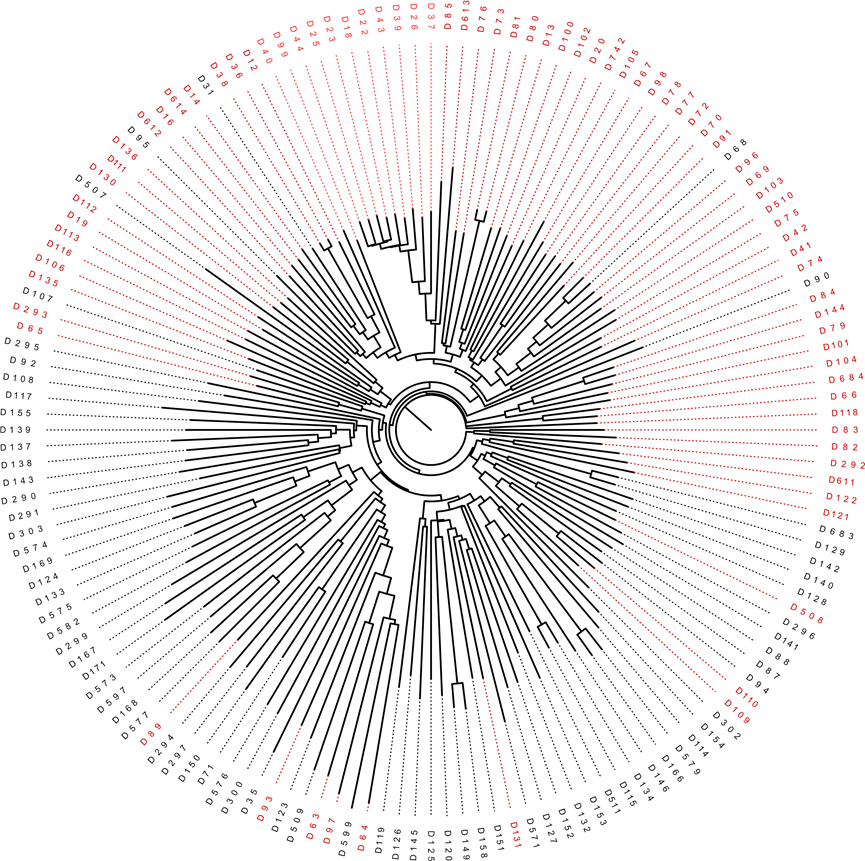


**Figure S4.** Dendrogram of 152 Chinese Yellow and Huai River Valleys Wheat Zone landraces estimated by shared-allele genetic distance using high-density DArT-seq and SSR markers. Cluster analysis was based on the neighbor-joining algorithm. Accessions have been assigned colors based on STRUCTURE v.2.3.4. analysis at *K* = 2. Red=group II, while the rest are group I (Black).
