# Supplementary material for: Genome-Wide Association Study for Adult-Plant Resistance to Stripe Rust in Chinese Wheat Landraces (Triticum aestivum L.) From the Yellow and Huai River Valleys
Source: Front Plant Sci. 2019 May 16;10:596. doi: 10.3389/fpls.2019.00596 (PMC6532019; doi:10.3389/fpls.2019.00596)
Supplement: Supplementary file 10 [file Data_Sheet_5.docx]

**
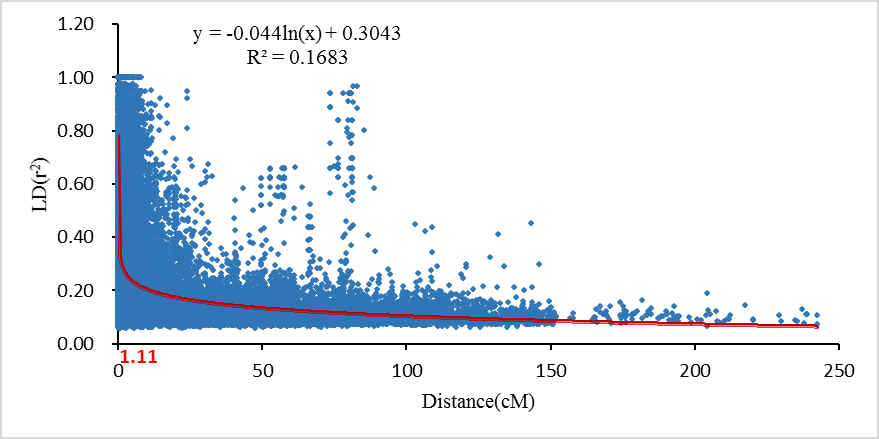
**


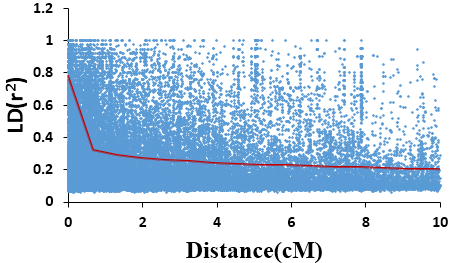


**Figure S5.** Scatterplot estimated the r^2^ for pairs of 7746 DArT-seq and SSR markers across chromosomes and genomes, showing LD decay, as measured the pairs of polymorphic marker loci by r^2^ against genetic distance (cM). The decay curves were plotted with predicted LD values. *Inset panel* provided much more details on LD decline for markers located within the first 10 cM.
